# Exploring the relationship between hypoxia in lung adenocarcinoma and tumor microenvironment immune cell infiltration

**DOI:** 10.1007/s12672-025-02822-7

**Published:** 2025-06-04

**Authors:** Changcong Gu, Shuai Guo, Haoxiang Li

**Affiliations:** 1 Department of Radiology, The First Hospital of Qinhuangdao, Qinhuangdao, Hebei China; 2 Department of Ultrasound, The First Hospital of Qinhuangdao, Qinhuangdao, Hebei China; 3 Department of Radiology, Guangdong Province, Guangdong Provincial People’s Hospital, Zhuhai Hospital (Jinwan Central Hospital of Zhuhai), Zhuhai, China

**Keywords:** Lung adenocarcinoma, Hypoxia, Prognosis, Immune gene

## Abstract

**Background:**

The hypoxic microenvironment affects the development of many types of tumors. It also triggers a series of immune response, and affects the level of immune cells infiltration. Here, the study aims to develop a gene marker based on hypoxia for prognosis evaluation in lung adenocarcinoma (LUAD), and investigate the relationship between hypoxia and immune cell infiltration in the tumor microenvironment (TME).

**Method:**

Retrieving LUAD cases from The Cancer Genome Atlas (TCGA) and Gene Expression Omnibus (GEO) databases. Through bioinformatics analysis, we screened out the hypoxia genes correlated with the prognosis of LUAD. A hypoxia risk score model was established by using gene expression level and expression coefficient. Using the median risk score value, we divided the patients in the two databases into high-risk and low-risk groups. The hypoxic model was then validated using survival analysis and receiver operating characteristic (ROC) curves. The CIBERSORT calculation method was employed to analyze the infiltration of immune cells. Finally, we analyzed the correlation between immune genes and hypoxia.

**Result:**

Patients in the high-risk group, characterized by higher hypoxia risk scores, exhibited significantly poorer prognosis compared to those in the low-risk group. In addition, we found significant differences in the infiltration rates of these five types of immune cells (M0 macrophages, M1 macrophages, resting mast cells, activated mast cells, monocytes, resting NK cells and activated CD4^+^ memory T cells) in both groups of tissues. After screening, the expression levels of the four immune genes (CCR7, CXCL10, CXCL11, and CCL19) were significantly associated with hypoxia risk in both groups.

**Conclusion:**

We discovered that the hypoxia risk score was related to prognosis and immune cell infiltration rate of LUAD. This finding may provide new ideas for LUAD immunotherapy.

## Introduction

Lung cancer is the most frequently diagnosed cancer worldwide and the leading cause of cancer-related death [1]. Lung adenocarcinoma is the most common subgroups of NSCLC which accounts for the majority of lung tumors [2]. Although there were substantial advances in early diagnosis and care for lung cancer, the patient survival is still rather low [3]. Therefore, investigating underlying mechanism of LUAD is necessary. As one of the important characteristics of TME, hypoxia play many crucial roles in the development and metastasis of tumors [4]. The growth and invasion of tumor need more metabolic requirements and higher oxygen consumption, resulting in hypoxia microenvironment [5]. Hypoxia-inducible factors (HIFs) have been shown to regulate the expression of many genes involved in metabolic control, angiogenesis, and apoptosis, as well as medication and drug resistance in solid tumors [6–8]. New evidence shows that hypoxia can limit the immune system and inhibit effective immunity [9, 10]. Hypoxia can influence immune cell expression in TME, resulting in the release of immunostimulatory factors [11, 12]. All this evidence suggests that Immune cells and hypoxia are intimately connected.

Therefore, detecting the immune cell is crucial. Currently, common clinical detection methods include immunohistochemistry (IHC) and flow cytometry (FACS), but these methods have certain limitations, such as complex procedures and low sensitivity. However, in recent years, researchers have proposed more advanced and effective detection methods. For example, the integrated Machine Learning and Genetic Algorithm‐driven Multiomics analysis (iMLGAM) method proposed by Ye et al., which combines machine learning and genetic algorithms, utilizes multi-omics data for predicting immunotherapy responses, significantly improving the accuracy of immune gene detection [13]. Zhang et al. developed a post-transcriptional modification learning signature (PTMLS) that analyzes the immune characteristics and tumor microenvironment of LUAD patients, providing more accurate prognostic predictions and aiding in the personalized selection of immunotherapies [14]. Ye et al. further explored the immune microenvironment through single-cell RNA sequencing (scRNA-Seq) and found that the distribution and functional state of specific immune cell populations were closely associated with immunotherapy outcomes [15]. Furthermore, in the treatment of these genes, nanotechnology has become a research hotspot, attracting significant attention from researchers. It achieves precise targeting of lung cancer tumors through nanoparticles and regulates immune evasion factors (such as B4GALT2), thereby enhancing the efficacy of immunotherapy [16].

Given the pivotal role of hypoxia in modulating the immune landscape within the tumor microenvironment, elucidating the interplay between hypoxia and immune cell infiltration is crucial for the advancement of therapeutic strategies. In this context, This study used the TCGA, GEO and MSigDB databases to construct and validate a hypoxia risk model for LUAD, and then used the risk score for hypoxia as a breakthrough to inquire into the differences in the rate of immune cell infiltration to help assess the status of hypoxia and the immune microenvironment, which may have applications in guiding the use of selective therapies.

## Materials and methods

### Data collection

RNAseq count data and clinical data obtained from the TCGA and the GEO(GSE68465) database. The data is organized by Strawberry Perl. Hypoxia related genes (HRG) were collected from the Gene Set Enrichment Analysis (GSEA) database. The Search Tool for the Retrieval of Interacting Genes/Proteins (STRING) was used to construct the hypoxia gene interaction network. R software is used to sort and filter according to the number of adjacent nodes.

### Construction of hypoxia model

Screening and identifying hypoxia genes independently were related to the prognosis of LUAD. We first computed the risk scores for each patient in both databases, and then classified the cases into high and low risk groups depending on the average risk score for subsequent evaluation.

### Survival analysis

A total of 522 and 444 specimens were collected from TCGA and GEO databases. After excluding samples with missing survival data and samples from normal tissue, the total number of participants from the TCGA and GEO databases is corrected to 504 and 442, respectively. Tables 1 and 2 showed the detailed demographic characteristics. We performed survival analysis for these data with the prognostic model by using the R “survminer” and"survival"package. Actuarial survival curves were generated using the Kaplan–Meier method and the log-rank test was used to evaluate differences between them. The survival curve was constructed using the Kaplan–Meier method, and the difference was assessed by log-rank test. P-values ≤ 0.05 were regarded as statistically significant.

**Table 1:** The characteristics of patients in TCGA

Parameter	Subtype	No	Percent (%)
Age (years)	≥ 60	359	71.23
	< 60	135	26.79
	No report	10	1.98
Gender	Male	235	46.63
	Female	269	53.37
Survival status	Alive	321	63.69
	Dead	183	36.31
TNM staging			
	T1	168	33.33
	T2	270	53.57
	T3	45	8.93
	T4	18	3.57
	TX	3	0.60
	N0	324	64.28
	N1	95	18.85
	N2	71	14.09
	N3	2	0.40
	NX	12	2.38
	M0	336	66.67
	M1	25	4.96
	MX	143	28.37
Total		504

**Table 2:** The characteristics of patients in GSE68465

Parameter	Subtype	No	Percent (%)
Age(years)	≥ 60	314	71.04
	< 60	128	28.96
Gender	Male	223	50.45
	Female	219	49.55
Survival status	Alive	206	46.61
	Dead	236	53.39
TNM staging			
	T1	150	33.94
	T2	251	56.79
	T3	28	6.33
	T4	11	2.49
	TX	2	0.45
	N0	299	67.65
	N1	87	19.68
	N2	53	11.99
	NX	3	0.68
Total		442	

### Cox regression analysis

Univariate Cox regression analysis was performed to screen hypoxia-related genes (HRGs) linked to prognosis (P < 0.05). Selected genes underwent multivariate Cox regression analysis, where a Cox proportional hazards model was fitted using maximum likelihood estimation (MLE) based on gene expression data. Regression coefficients (β) were calculated to assess their prognostic contribution, and a risk score was derived for each patient. Additionally, univariate and multivariate Cox analyses evaluated prognostic factors including age, gender, risk score, and TNM stage, with the latter adjusting for covariates.

### ROC curve analysis

We performed ROC curve analysis based on “survival”, “survminer”, and “survival ROC” packages in R statistical language (Version R 4.0.4). The 1 -, 2 -, and 3-year survival rates were assessed and recorded for patients in both databases. The value under the ROC curve is over 0.5 as the threshold for the model to predict the survival rate accurately. The survival time of each group was expressed by risk curve and risk columns.

### Relationship between differential gene-expression and risk score

The Genes which play an important role in the regulation of immune cells were identified, and then we investigated the correlations of differential gene-expression and risk score between the patients of high and low risk groups.

### Gene set enrichment analysis

GSEA software v 4.1.0 (http://www.broadinstitute.org/gsea) was used to analysis the genetic information of all tumor samples. False discovery rate (FDR) q-values < 0.05 and nominal P values < 0.05 was considered significant.

## Results

### Screening hypoxia related genes and constructing hypoxia model

**Fig. 1:**
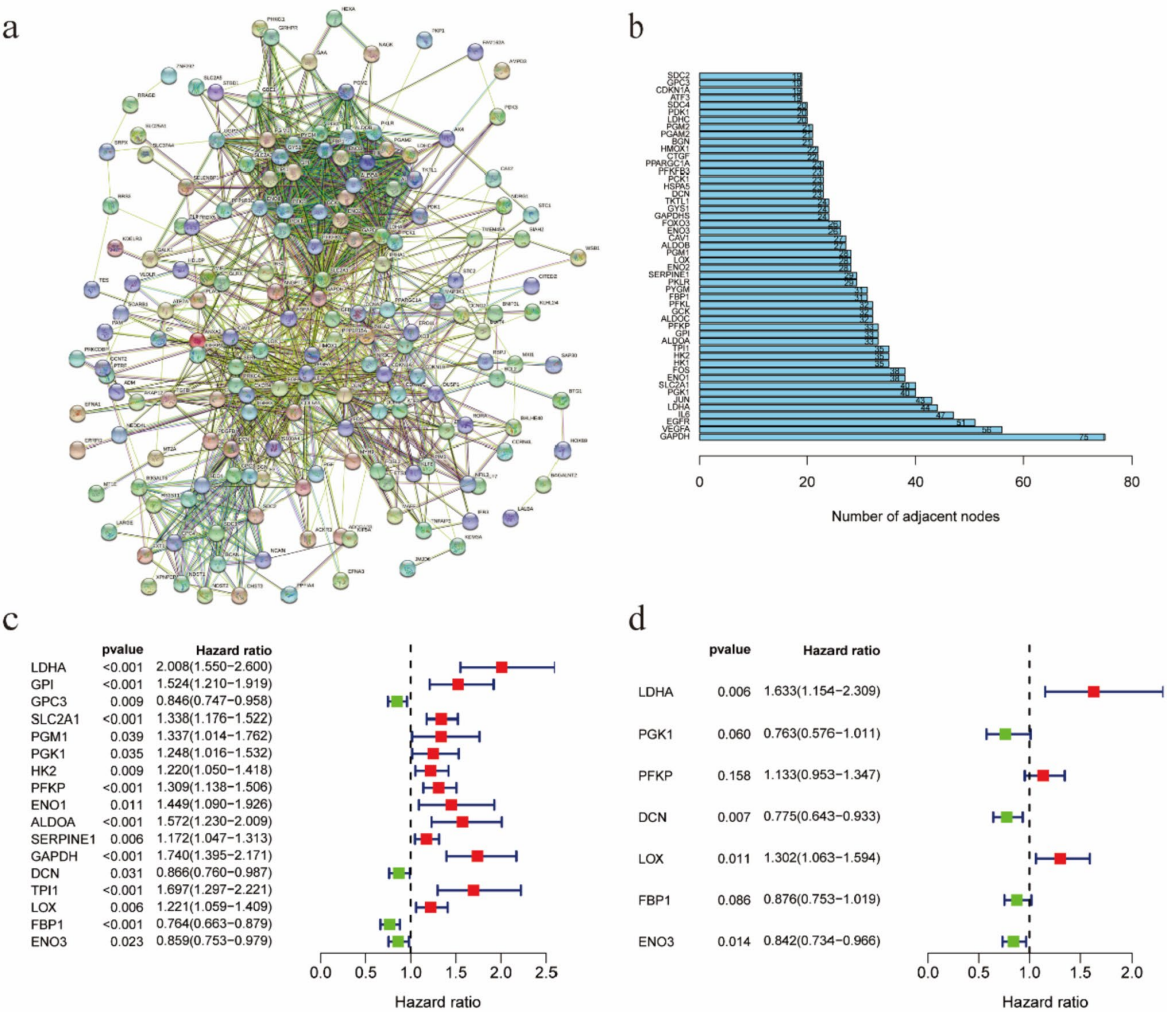
Relationship between hypoxia related genes and prognosis of patients. **a** The protein–protein interaction network of hypoxia-related genes. **b **The top 50 genes selected based on the number of nodes and their sub-nodes. **c** Univariate Cox regression analysis screened candidate genes with P-values < 0.05. **d** Among the prognostic genes of lung adenocarcinoma, the gene displayed is the one related to prognosis in a multifactor prognostic model of hypoxia

To constructing hypoxia model, we first screened out all HRG collected in the Gene Set Enrichment Analysis (GSEA) database, and then constructed the protein–protein interaction (PPI) Network of the extracted hypoxia gene list from the STRING database(Fig. 1a). Furtherly, we identified the 50 genes with the most significant interactions (Fig. 1b). We used TCGA-LUAD database to extract the gene expression data and clinical information, and then screened out prognosis related hypoxia genes by using univariate Cox regression analysis (Fig. 1c). Subsequently, we applied multivariate Cox regression with stepwise selection based on the Akaike Information Criterion (AIC) to select the optimal subset of genes, balancing model fit and complexity, resulting in seven genes for the hypoxia risk model (Fig. 1d). These seven genes (LDHA, PGK1, PFKP, DCN, LOX, FBP1 and ENO3) had model coefficients of 0.4902, −0.2705, 0.1249, −0.2551, 0.2638, −0.1324 and −0.1722 accordingly.

### Relationship between hypoxia-related genes and prognosis

**Fig. 2:**
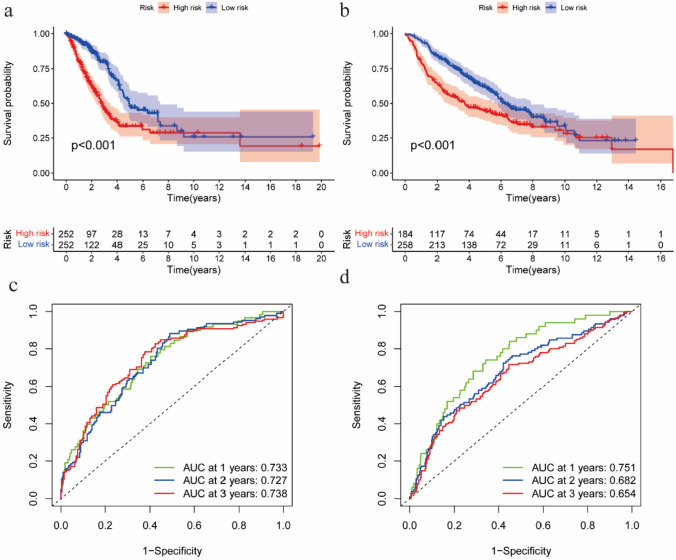
Survival analysis and multiple ROC curve results. **a**, **b** Kaplan–Meier survival curves for patients with lung adenocarcinoma in The Cancer Genome Atlas and Gene Expression Omnibus databases. **c**, **d** Receiver operating characteristic curve analysis model prediction accuracy

**Fig. 3:**
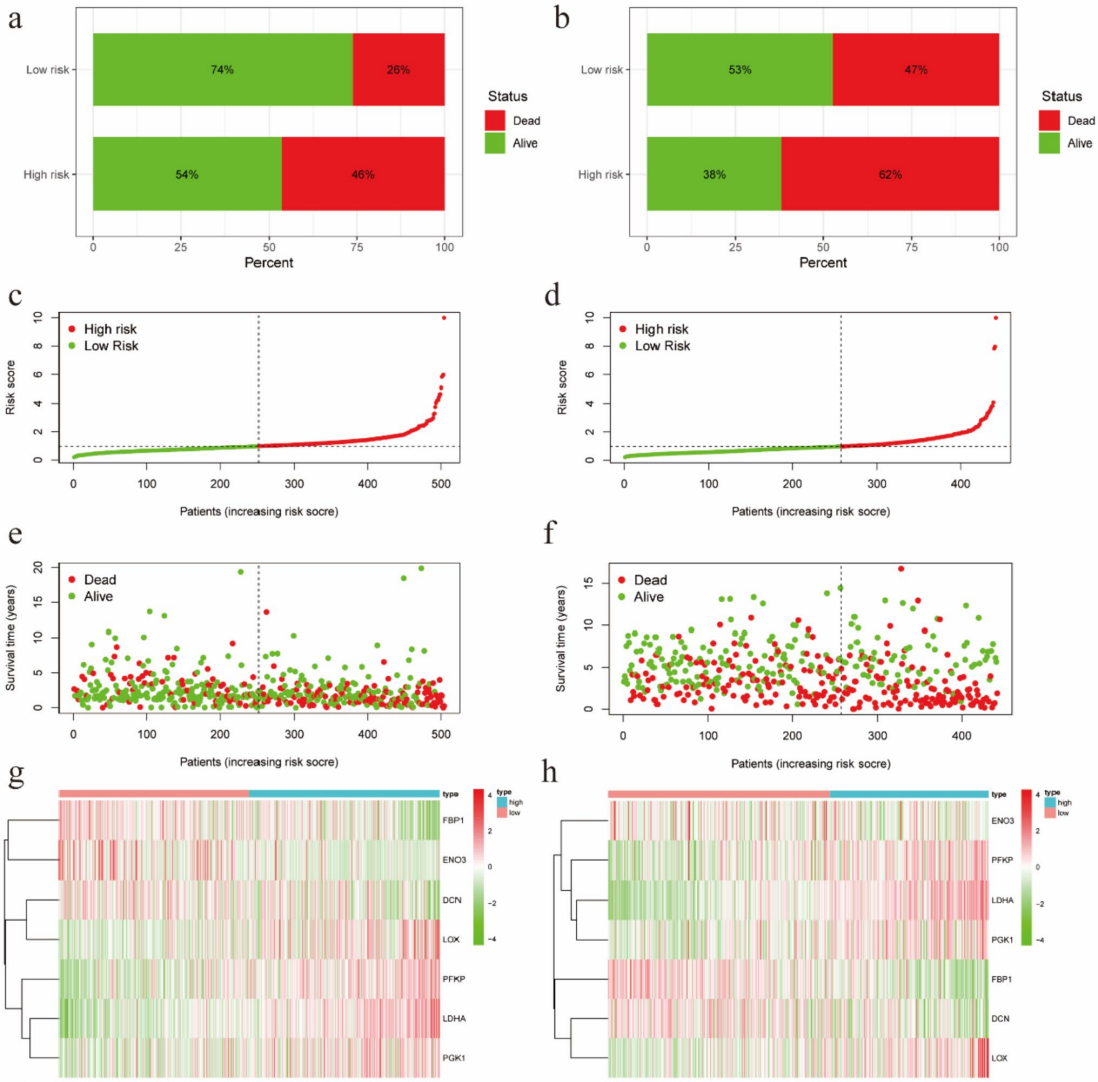
Risk prediction and hypoxia gene expression in hypoxia model. **a**, **b** Patient survival rate in TCGA and GEO databases. **c**, **d** Patient risk scores in TCGA and GEO databases. **e**, **f** The survival rate of patients in high and low risk groups in TCGA and GEO databases. **g**, **h** Heat maps of gene expression levels in hypoxia model for high and low risk groups in TCGA and GEO databases

The risk score for each patient in both databases = ∑gene expression * coefficient. Based on the median risk score derived from the TCGA cohort, 504 patients were equally divided into low- and high-risk subgroups, with 252 patients in each group. Similarly, based on the median risk score established in the TCGA cohort, 442 patients in the GEO cohort were classified, with 258 patients assigned to the low-risk group and 184 patients assigned to the high-risk group. Survival analysis showed significant differences between the low- and high-risk groups in both databases (P < 0.05; Fig. 2a, b). Additionally, to assess the accuracy of the model for survival estimates, the ROC curves in both databases were constructed. The results show that the AUCs for the 1-, 2-, and 3-year survival rates were all greater than 0.65(Fig. 2c, d), which indicates that the hypoxia model has accurately predictive ability. To visually illustrate the difference in patient survival between the high-risk and low-risk groups in both database, we made a risk histogram. Figure 3a, b demonstrated that patients in high-risk group had a lower probability of survival and those in the other group had a higher probability of survival, meaning that hypoxia model can adequately discriminate between high- and low-risk patients. In addition to illustrate the survival status and corresponding risk score for both groups of patients, we made the risk curve and scatterplot. Figure 3c–f displayed that the lower the risk score was, the more the survival rate. Finally, to show the expression difference of these 7 HRG in our model, we used a heat-map plot. Figure 3g, h revealed that in the high-risk community, LDHA, PFKP, LOX and PGK1 were strongly expressed, while in the low-risk group, DNC, FBP1, and ENO3 were upregulated. Finally, to reveal the difference between high- and low-risk groups, we performed GSEA with the downloaded GSEA software (www.broadinstitute.org/gsea). We found that the genes set of the high-risk group in the TCGA database were significantly enriched in G2M checkpoints, glycolysis, E2 F signaling pathway, MYC signaling pathway, MTORC1 signaling pathway, mitotic spindle and unfolded protein reactions compared to the low-risk group (Table 3). These results were further verified in the GSE68465 database (Table 4).

**Table 3:** Pathway enrichment in the group of patients at high risk of hypoxia in The Cancer Genome Atlas database

Name	Size	ES	NES	NOM p-val	FDR q-val	FWER p-val
HALLMARK_GLYCOLYSIS	199	0.67	2.53	0	0	0
HALLMARK_HYPOXIA	200	0.63	2.44	0	0	0
HALLMARK_MTORC1_SIGNALING	200	0.71	2.41	0	0	0
HALLMARK_G2M_CHECKPOINT	200	0.83	2.34	0	0	0.001
HALLMARK_E2 F_TARGETS	200	0.85	2.22	0	0.001	0.002
HALLMARK_MYC_TARGETS_V1	200	0.79	2.22	0	0.001	0.002
HALLMARK_MITOTIC_SPINDLE	199	0.64	2.2	0	0.001	0.003
HALLMARK_UNFOLDED_PROTEIN_RESPONSE	113	0.59	2.07	0	0.004	0.02
HALLMARK_EPITHELIAL_MESENCHYMAL_TRANSITION	200	0.69	2.07	0.004	0.004	0.022
HALLMARK_PI3 K_AKT_MTOR_SIGNALING	105	0.5	2	0	0.007	0.047
HALLMARK_MYC_TARGETS_V2	58	0.75	1.95	0.006	0.012	0.075
HALLMARK_ANGIOGENESIS	36	0.62	1.9	0.002	0.018	0.11
HALLMARK_APICAL_SURFACE	44	0.56	1.87	0.006	0.02	0.128
HALLMARK_DNA_REPAIR	149	0.52	1.84	0.007	0.023	0.147
HALLMARK_TNFA_SIGNALING_VIA_NFKB	200	0.53	1.79	0.026	0.032	0.187
HALLMARK_APICAL_JUNCTION	199	0.47	1.77	0.022	0.035	0.211
HALLMARK_PROTEIN_SECRETION	96	0.5	1.76	0.031	0.035	0.219
HALLMARK_ANDROGEN_RESPONSE	100	0.44	1.75	0.006	0.036	0.236
HALLMARK_TGF_BETA_SIGNALING	54	0.52	1.74	0.034	0.038	0.256
HALLMARK_UV_RESPONSE_UP	158	0.42	1.68	0.004	0.049	0.314
HALLMARK_APOPTOSIS	161	0.42	1.68	0.014	0.047	0.319
HALLMARK_ESTROGEN_RESPONSE_LATE	199	0.39	1.62	0.004	0.061	0.383
HALLMARK_INFLAMMATORY_RESPONSE	200	0.45	1.59	0.074	0.069	0.423
HALLMARK_IL2_STAT5_SIGNALING	199	0.37	1.57	0.052	0.073	0.446
HALLMARK_ESTROGEN_RESPONSE_EARLY	200	0.38	1.56	0.014	0.072	0.453
HALLMARK_SPERMATOGENESIS	135	0.41	1.55	0.041	0.072	0.462
HALLMARK_COMPLEMENT	200	0.4	1.51	0.076	0.085	0.511
HALLMARK_REACTIVE_OXYGEN_SPECIES_PATHWAY	49	0.49	1.5	0.105	0.087	0.516
HALLMARK_KRAS_SIGNALING_UP	200	0.36	1.45	0.072	0.104	0.572
HALLMARK_INTERFERON_GAMMA_RESPONSE	200	0.46	1.43	0.184	0.112	0.605
HALLMARK_CHOLESTEROL_HOMEOSTASIS	74	0.4	1.41	0.113	0.116	0.623
HALLMARK_UV_RESPONSE_DN	144	0.37	1.4	0.12	0.118	0.632
HALLMARK_IL6_JAK_STAT3_SIGNALING	87	0.41	1.38	0.15	0.122	0.65
HALLMARK_INTERFERON_ALPHA_RESPONSE	97	0.47	1.31	0.254	0.157	0.709
HALLMARK_COAGULATION	138	0.32	1.18	0.236	0.252	0.839
HALLMARK_NOTCH_SIGNALING	32	0.36	1.18	0.241	0.246	0.842
HALLMARK_HEDGEHOG_SIGNALING	36	0.37	1.17	0.292	0.25	0.85
HALLMARK_ALLOGRAFT_REJECTION	200	0.33	1.08	0.393	0.323	0.904
HALLMARK_WNT_BETA_CATENIN_SIGNALING	42	0.32	1.03	0.406	0.371	0.929
HALLMARK_OXIDATIVE_PHOSPHORYLATION	200	0.29	0.85	0.589	0.593	0.984

**Table 4:** Pathway enrichment in the group of patients at high risk of hypoxia in the Gene Expression Omnibus database

Name	Size	ES	NES	NOM p-val	FDR q-val	FWER p-val
HALLMARK_G2M_CHECKPOINT	185	0.74	2.25	0	0	0
HALLMARK_E2 F_TARGETS	182	0.78	2.17	0	0.002	0.003
HALLMARK_MTORC1_SIGNALING	188	0.63	2.17	0	0.001	0.003
HALLMARK_MYC_TARGETS_V1	184	0.73	2.12	0	0.001	0.004
HALLMARK_GLYCOLYSIS	183	0.5	2.04	0	0.004	0.013
HALLMARK_MITOTIC_SPINDLE	180	0.51	1.97	0.002	0.008	0.03
HALLMARK_UNFOLDED_PROTEIN_RESPONSE	102	0.52	1.94	0.002	0.009	0.037
HALLMARK_MYC_TARGETS_V2	55	0.67	1.91	0.008	0.011	0.051
HALLMARK_HYPOXIA	184	0.4	1.61	0.019	0.1	0.376
HALLMARK_DNA_REPAIR	133	0.43	1.61	0.028	0.092	0.387
HALLMARK_PI3 K_AKT_MTOR_SIGNALING	99	0.36	1.55	0.012	0.116	0.487
HALLMARK_SPERMATOGENESIS	122	0.4	1.53	0.036	0.12	0.529
HALLMARK_PROTEIN_SECRETION	94	0.44	1.52	0.056	0.112	0.531
HALLMARK_ANGIOGENESIS	35	0.44	1.4	0.104	0.191	0.743
HALLMARK_OXIDATIVE_PHOSPHORYLATION	179	0.42	1.31	0.202	0.269	0.862
HALLMARK_ANDROGEN_RESPONSE	93	0.35	1.28	0.169	0.293	0.902
HALLMARK_ESTROGEN_RESPONSE_LATE	195	0.25	1.15	0.194	0.462	0.975
HALLMARK_UV_RESPONSE_UP	151	0.25	1.1	0.271	0.507	0.987
HALLMARK_TNFA_SIGNALING_VIA_NFKB	193	0.28	0.93	0.513	0.818	1
HALLMARK_WNT_BETA_CATENIN_SIGNALING	37	0.25	0.91	0.588	0.808	1
HALLMARK_REACTIVE_OXYGEN_SPECIES_PATHWAY	43	0.27	0.87	0.612	0.856	1
HALLMARK_TGF_BETA_SIGNALING	51	0.25	0.85	0.657	0.855	1
HALLMARK_EPITHELIAL_MESENCHYMAL_TRANSITION	189	0.27	0.82	0.597	0.867	1
HALLMARK_CHOLESTEROL_HOMEOSTASIS	60	0.22	0.8	0.7	0.873	1
HALLMARK_APICAL_SURFACE	33	0.22	0.79	0.809	0.856	1
HALLMARK_COMPLEMENT	185	0.19	0.72	0.845	0.929	1
HALLMARK_INFLAMMATORY_RESPONSE	193	0.19	0.67	0.826	0.958	1
HALLMARK_INTERFERON_GAMMA_RESPONSE	174	0.19	0.6	0.837	0.976	1
HALLMARK_INTERFERON_ALPHA_RESPONSE	78	0.21	0.59	0.84	0.946	1

### Influence of clinical features on prognosis of lung adenocarcinoma

**Fig. 4:**
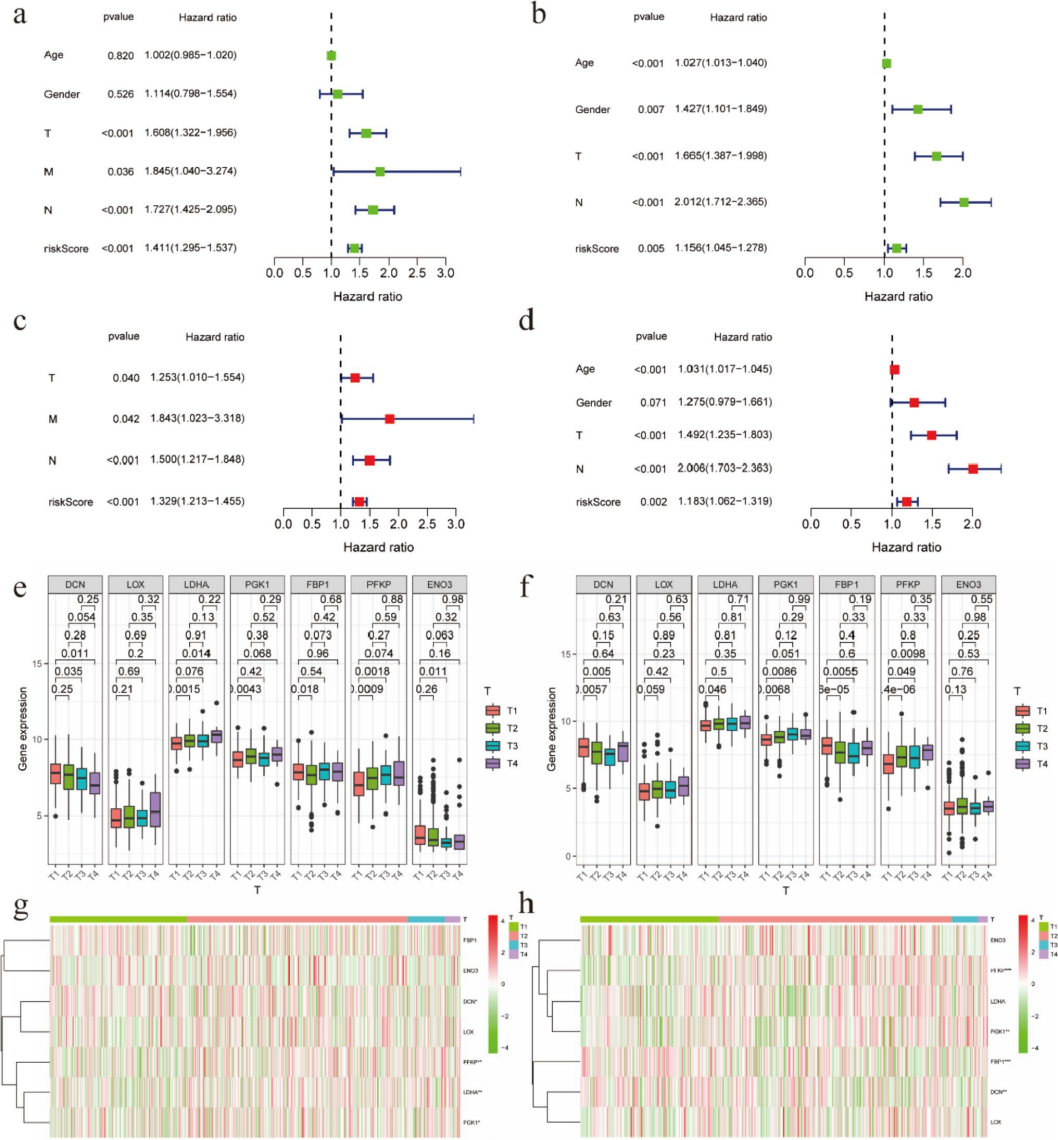
The relationship between hypoxia model and clinical factors. **a**, **b** Univariate analysis of prognosis. **c**, **d** Multivariate analysis of prognosis. **e**, **f** Comparison of expression levels of multiple genes at different T stages in the hypoxia models. **g**, **h** Heat maps of expression levels of genes at different T stages in the hypoxia models

Various clinical features have different impact on the prognosis of cancer patients. Thus, to assess the prognostic value of the risk score more correctly, we examined clinical characteristics including age, gender and TNM stage in Prognostic analysis both in the TCGA and GEO database. We found that in the TCGA database, univariate Cox regression analysis of clinical features revealed that prognosis was significantly associated with TNM stage (P < 0.05) and risk score (P < 0.001), while prognosis was no significantly associated with age and gender (Fig. 4a). The significant variables were then used in the multivariate Cox analysis. Figure 4c showed that T stage (P = 0.04), N stage (P < 0.001), and risk score (P < 0.001) were shown to be independent prognostic variables. Furthermore, we observed that the p value of age, T stage, N stage and risk score in univariate and multivariable Cox analysis were all less than 0.05, indicating that these factors were independent prognostic variables In the GEO database (Fig. 4b, d). Then we observed differences in the seven hypoxia genes expression at different T stages in both databases. Figure 4e–h revealed that the LDHA expression level was statistically significant in the TCGA database, while the PFKP expression level was statistically significant in the GEO database.

### Immune cells infiltration analysis

**Fig. 5:**
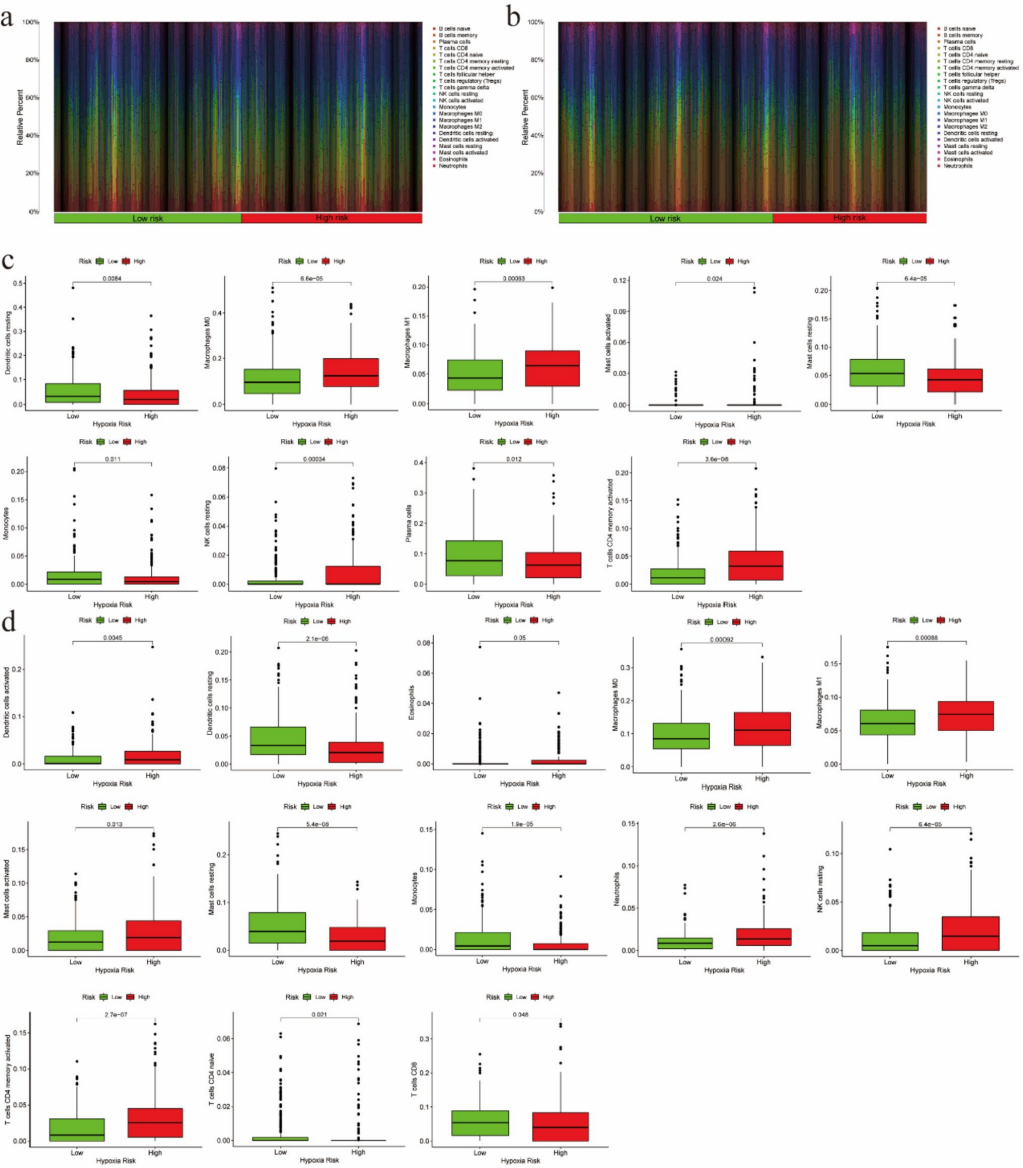
The infiltration of immune cells. **a**, **b** Heat maps of hypoxia risk and immune cell infiltration in TCGA and GEO databases. **c**, **d** immune cell infiltration was significantly associated with the risk of hypoxia in TCGA and GEO databases (P < 0.05)

**Fig. 6:**
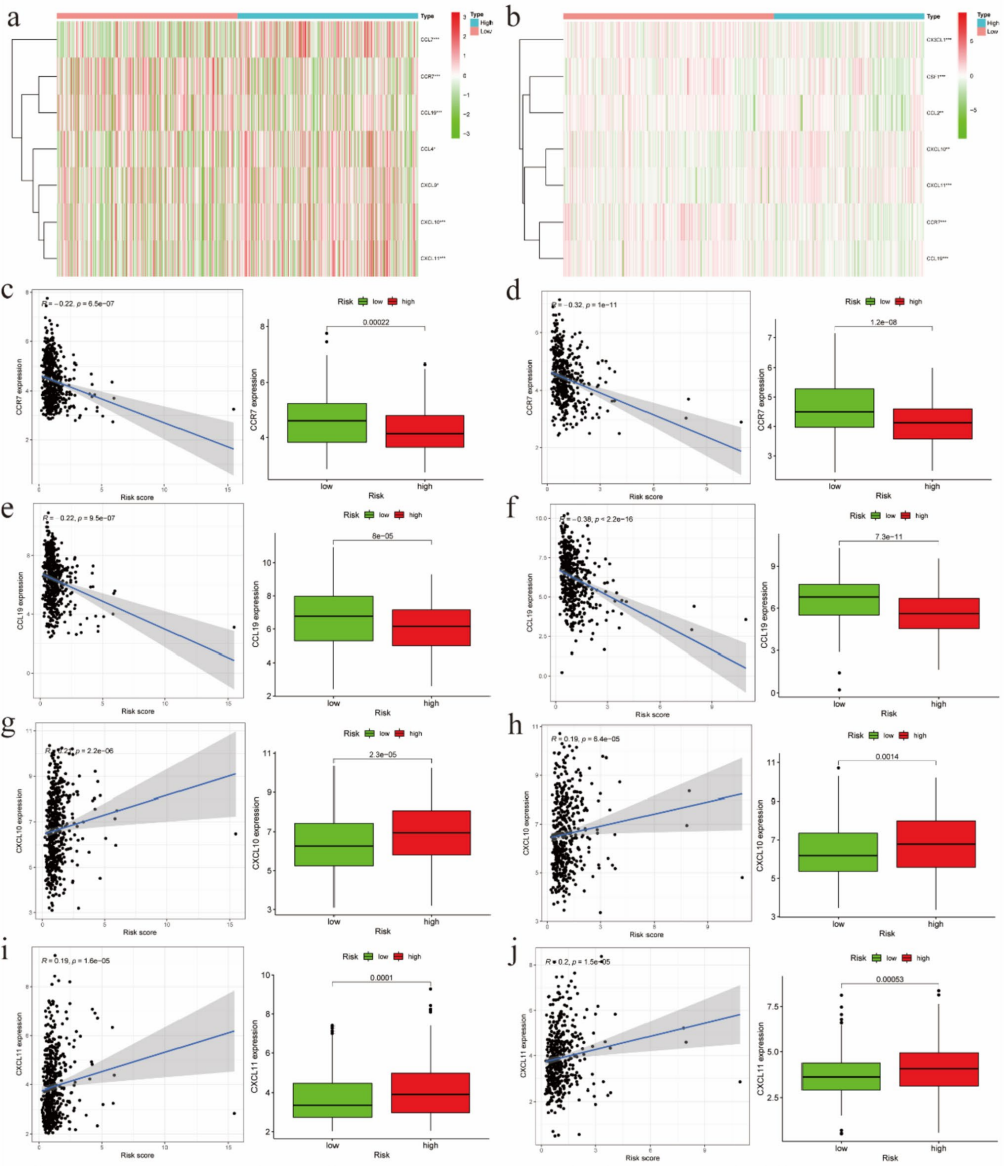
Relationship between genes regulating immune cell behavior and risk of hypoxia. **a**, **b **Heat map showing the expression levels of related regulatory genes of activated immune cells in different hypoxia risk groups(**P < 0.01; ***P < 0.001). **c**–**j** Scatter plots showing the correlation between the expression of four genes from TCGA and GEO databases with immune cell regulation, displaying differences in the expression levels between high and low risk groups (P < 0.05)

A hypoxic microenvironment had been reported to affect anti-tumor immune responses and promote immune escape [6]. Thus, we estimated that there were 22 immune cell types in the two databases. Thus, we used CIBERSORT to calculate the rate of 22 immune cells infiltration and used the heat map to display the infiltration of each risk group (Fig. 5a, b). Figure 5c, d shows significantly differences in the infiltration ratio of 9 and 13 types of immune cells between the high- and low-risk groups in TCGA and GEO databases, respectively. Among them, the infiltration rates of seven immune cells (M0 macrophages, M1 macrophages, resting mast cells, activated mast cells, monocytes, resting NK cells and activated CD4 + memory T cells) were significantly different between the lower- and higher-risk groups in the two databases (P < 0.05). To identify the immune-related genes that play a regulatory role in the regulation of macrophages, monocytes, NK cells and CD4 + T cells, we used the online platform of the Tracking Tumor Immunophenotype. We then created a heatmap to visualize the expression of these immune-related genes in the high- and low-risk groups in both databases. The results showed that the expression levels of CCR7, CCL19, CXCL10 and CXCL11 differed significantly across the high- and low-risk groups (Fig. 6a, b; P < 0.05). Figure 6c–j provides the correlation curves of the correlation between the expression levels of these four genes and the risk scores. We found that the risk scores were positively correlated with the expression levels of CXCL10 and CXCL11 and negatively correlated with the expression levels of CCR7 and CCL19. We also discovered that the expression levels of these four genes differed between the low‐ and high‐risk groups.

## Discussion

Hypoxia is a characteristic of TME, which is associated with tumor survival, proliferation, invasion and drug tolerance by acting on the appropriate pathway or regulating immune cells [17–20]. In addition, hypoxia plays a crucial role in influencing an immune anticancer response [10]. Hypoxia inducible factor participates in carcinogenesis and tumor progression by controlling genes involved in glycolysis, angiogenesis and metabolism [21]. In this study, we screened genes related to hypoxia in LUAD, and identified seven core genes (LDHA, PGK1, PFKP, DCN, LOX, FBP1 and ENO3) that were significantly related to prognosis. Previous studies have shown that Previous studies have shown that PGK1 is associated with the development and progression of many types of cancers, as well as chemoradiotherapy resistance [22, 23]. Invasion and transfer of non-small cell lung cancer may be inhibited by DCN through the TGF-β signaling pathway [24]. FBP1 has been identified as an oncoprotein and is overexpressed in many types of malignant tumors. Some studies have found that FBP1 can accelerate cell cycle transition and metastasis, thereby promoting the development of ovarian cancer [25]. Moreover, FBP1 was reported to participate in epithelial mesenchymal metastasis, invasion and transition metastasis of prostate cancer cells by regulating MAPK signaling pathway[26]. The oncogenic role of LDHA has previously been reported in numerous cancers, which can promote the malignant progression of tumor by increasing lactate production, accelerating glucose uptake and regulating several cancer-related molecules [27, 28]. PFKP promotes the malignant progression of OSCC by regulating starvation-mediated autophagy, glycolysis and EMT [29]. Multiple studies have suggested that many stages of cancer development including primary growth, angiogenesis, metastasis, and invasion are influenced by LOX [30]. Some studies show that ENO3 has over expression and selective anticancer effect in STK11 Mutant Lung Cancers [31]. These results indicate that the expression levels of HRG are strongly associated with tumor development and metabolism [32]. Therefore, we used these genes to establish HRG models.

Through the relevant research on the mechanism of hypoxia, people found that it has a great impact on the prognosis of tumor [33, 34]. To explore the association between the prognosis of LUAD and HRG, we used the multiplication of coefficients and the expression levels of these seven HRG (LDHA, PGK1, PFKP, DCN, LOX, FBP1 and ENO3) as the risk score to estimate patient prognosis In databases from the GEO and TCGA. The results demonstrated that Patient prognosis was significantly different between high- and low-risk group, and the patient survival in the high-risk group was significantly reduced. Moreover, ROC curves in the TCGA and GEO datasets showed that high accuracy of the hypoxia risk model.

In addition, we investigated the impact of other factors on patient prognosis, such as age, gender, TNM stage, and risk score. The conclusion is that the risk score corresponds to patient prognosis. The results of univariate and multivariate prognostic analysis in the two databases are statistically significant, and the risk score can be an independent prognostic indicator. Additionally, to further investigate the role of hypoxia-related genes (HRGs) in tumor progression, we analyzed their expression in T staging within a hypoxia model (Fig. 4e, f). Statistical analysis revealed significant expression differences for most HRGs (p < 0.05), highlighting their dynamic roles in the tumor microenvironment (TME) under hypoxic conditions. Notably, LDHA, PGK1, and PFKP showed elevated expression in later T stages (T3 and T4). This upregulation suggests their key involvement in metabolic adaptation to hypoxia, enhancing lactate production and glycolysis—critical survival mechanisms for tumor cells in solid tumors [28, 35, 36]. In contrast, DCN, FBP1, and ENO3 exhibited variable expression patterns, with significant differences between T stages, which may be related to extracellular matrix remodeling and metastasis [24, 37]. Although LOX showed no significant expression differences across T stages, it may indirectly affect tumor growth and invasiveness by altering the extracellular matrix surrounding the tumor [38]. These findings emphasize the prognostic potential of HRGs in LUAD and their stage-specific contributions to tumor behavior. Future studies should validate these patterns in larger cohorts and explore therapeutic targeting of these genes, such as LDHA inhibitors, to disrupt hypoxic adaptation and improve LUAD outcomes.

We then applied GSEA to identify the significantly enriched pathways in the high-risk group from two databases. We found that most of the enriched pathways were related to tumor proliferation, cell cycle dysregulation, and metabolic reprogramming, particularly glycolysis. The HRG genes, LDHA, PGK1, and PFKP, play key roles in these processes. LDHA promotes lactate production to support the energy demands under hypoxia, while acidifying the microenvironment to promote tumor invasion [39]. In addition to glycolysis, PGK1 also contributes to nucleotide synthesis and redox balance, directly promoting tumor proliferation [40]. PFKP is a rate-limiting enzyme in glycolysis, regulating metabolic flux and influencing epithelial-mesenchymal transition (EMT) and autophagy, thereby enhancing tumor invasiveness [41]. These multifaceted roles highlight the complex relationship between hypoxia-induced metabolism and the malignant phenotype in the TME. Furthermore, the MTORC1 signaling pathway, enriched in the TCGA and GSE68465 databases, is a key regulator of cell growth and metabolism. Its activation in high-risk populations may support tumor proliferation and survival under hypoxic stress, leading to the observed poor prognosis [42]. These findings emphasize the key role of hypoxia in driving LUAD progression through glycolysis and MTORC1 signaling, providing a mechanistic explanation for the poor outcomes observed in high-risk patients. In terms of therapy, these insights suggest that targeting metabolic pathways, such as inhibiting LDHA or PFKP to disrupt glycolysis, or using MTORC1 inhibitors like rapamycin, may alleviate tumor growth and invasion [43]. Targeting these metabolic pathways provides a theoretical foundation for the development of new therapeutic strategies. Future research should focus on validating these targets in different populations and exploring how to integrate them into personalized treatment strategies for LUAD.

Many previous studies have explored the associations of immune cells and hypoxia in LUAD. One of them reported that GBE1 was implicated in regulation of PD-L1 in LUAD cells and recruitment of CD8 ^+^ T lymphocytes in TME through IFN-I/sting signaling pathway [44]. Another study showed that expression of PD-L1 in LUAD could be enhanced by EML4-ALK via the HIF-1 α and STAT3 pathways [45]. In this study, we investigated the differences of immune cell infiltration between the high-risk and low-risk groups in both databases and aimed to further investigate the potential connection between hypoxia and immune cell infiltration. The results revealed significant differences in the levels of infiltration of nine and thirteen types of immune cells from TCGA and GEO databases between the two groups, respectively. Additionally, the infiltration of M0 macrophages, M1 macrophages, resting mast cells, activated mast cells, monocytes, resting NK cells and activated CD4 + memory T cells were significantly different between the two risk groups from both databases. Then, we performed screening the genes regulating macrophages, monocytes, NK cells and CD4^+^ T cells identified that there were significant differences in the expression levels of CCR7, CXCL10, CXCL11, and CCL19 between the two risk groups.

The hypoxia-regulated TME in LUAD, quantified through the risk score model, may influence anti-tumor immune responses and promote immune escape. The high-risk group showed increased infiltration of immune cells (e.g., M0/M1 macrophages, and NK cells resting), suggesting an immunosuppressive TME, whereas the low-risk group exhibited reduced infiltration, possibly indicating enhanced immune surveillance. These differences highlight the key role of immune cell infiltration in assessing hypoxia-driven tumor progression and its prognostic impact. Further analysis revealed significant expression differences of immune-related genes (CCR7, CXCL10, CXCL11, CCL19) between risk groups, linking hypoxia to immune cell regulation. Notably, CXCL10 and CXCL11 were positively correlated with the risk score and are part of the CXCL9-11/CXCR3 axis, which is known to regulate immune cell migration and activation [46]. Their elevated expression in high-risk patients may reflect a compensatory response to hypoxia-induced immunosuppression, with potential as biomarkers for predicting the efficacy of immunotherapy. In contrast, CCR7 and CCL19, which were negatively correlated with the risk score, play roles in immune cell homing and anti-tumor immunity [47], suggesting that their downregulation in the high-risk group may impair effective immune responses. These findings provide new insights into the interaction between hypoxia and immune infiltration. CXCL10 and CXCL11 may serve as potential biomarkers for predicting immune therapy efficacy, while CCR7 and CCL19 may become new intervention targets to improve immune cell homing.

Among the 7 HRGs included in the hypoxia risk score model constructed in this study, these 6 genes (LDHA, PGK1, PFKP, DCN, LOX, FBP1) have been extensively reported in previous studies for their roles in tumor hypoxia response and progression. However, we note that research on ENO3 is relatively few, with existing studies mainly focusing on its overexpression and selective anticancer activity in STK11-mutant lung cancer [31]. Our study provides some confirmation of prior findings and expands on them by suggesting that ENO3 highly expressed in LUAD under hypoxic conditions, potentially contributing to tumor metabolism and immune regulation within the TME. This contribution may add a novel perspective to ENO3’s role in LUAD, warranting further investigation.

However, this study has several limitations. Notably, our research did not identify novel, unreported hypoxia-related genes, thus constraining the novelty of our gene selection. We plan to address this by integrating advanced genomic screening techniques to identify novel hypoxia-related genes in future studies. A key limitation is the lack of experimental validation for the associations between hypoxia-related risk scores, LUAD prognosis, and immune cell infiltration rates. We will actively seek experimental collaborations to continue this research. Additionally, given that the data used were obtained from public repositories and retrospective evaluations, the hypoxia-related risk profiles need further validation through multi-center studies to ensure their robustness. Moreover, TME cell infiltration shows a distinct distribution between the high-risk and low-risk groups; therefore, further exploration of the potential functions and mechanisms mediated by hypoxia-related risk genes is needed.

## Conclusion

Our findings indicate that hypoxia is an important factor within the TME. We identified genes associated with immune cell infiltration rates and observed their correlation with the hypoxia risk score. Specifically, our results suggest that HRG influence immune infiltration in LUAD, potentially enhancing the precision of clinical prognostic evaluations. The observed relationships between HRG, immune cells, and immune-related genes may offer insights relevant to tumor immunotherapy.

## Data Availability

The datasets analyzed in this study were obtained from publicly available repositories. The TCGA data are available at https://portal.gdc.cancer.gov/, and the GEO datasets are available at https://www.ncbi.nlm.nih.gov/geo/ under accession number GSE68465.
